# SIRT1 and exercise-induced bone metabolism: a regulatory nexus

**DOI:** 10.3389/fcell.2025.1522821

**Published:** 2025-03-26

**Authors:** Lijie Liu, Jiale Zhang, Runhong Cui, Na Wang, Yun Zhang, Lifei Liu, Xinan Zhang, Qingfeng Liu

**Affiliations:** ^1^ Department of Rehabilitation, Jinqiu Hospital of Liaoning Province, Shenyang, China; ^2^ College of Exercise and Health, Shenyang Sport University, Shenyang, China; ^3^ Department of General Surgery, Jinqiu Hospital of Liaoning Province, Shenyang, China

**Keywords:** SIRT1, exercise, bone health, osteoporosis, osteoblast, osteoclast

## Abstract

Regular exercise positively influences bone health, enhances bone density and strength, and reduces the risk of osteoporosis. Silent information regulator of transcription 1 (SIRT1) is a deacetylase that plays a pivotal role in the regulation of various biological processes. In this review, we explore the role of SIRT1 in modulating bone metabolism in response to exercise. SIRT1 regulates crucial cellular processes, including inflammation, aging, autophagy, and oxidative stress, in bone cells such as bone marrow mesenchymal stem cells, osteoblasts, and osteoclasts, in response to exercise-induced stimuli. Notably, exercise influences bone metabolism by modulating muscle metabolism and neurotransmitters, with SIRT1 acting as a key mediator. A comprehensive understanding of SIRT1’s regulatory mechanisms will facilitate a deeper exploration of the principles underlying exercise-induced improvements in bone metabolism, ultimately providing novel insights into the treatment of bone metabolic disorders.

## 1 Introduction

The skeleton is a dynamic structure that provides architectural support for posture control, and adapts to physical activity. Bones comprise a collagen matrix infused with calcium and phosphate deposits and various proteins, enabling it to store minerals and regulate nutrient and endocrine functions (Frost, 1994; Hart et al., 2020). Peak bone mass is achieved during young adulthood and declines with reduced physical activity, aging, glucocorticoids use, and metabolic diseases, increasing the risk of osteoporosis, which predisposes people to fragility fractures. This disease impairs mobility, prolongs bed rest in older patients, and even increases mortality (Tsugawa and Shiraki, 2020; Golden et al., 2014; Clynes et al., 2020).

Mechanical stimulation caused by exercise affects bone density. A lack of physical activity, such as from prolonged bed rest and microgravity, leads to insufficient mechanical stimulation and disrupts the balance between bone formation and resorption, thereby accelerating bone loss and exacerbating osteoporosis (D'Onofrio et al., 2023; Watson et al., 2018; Hu et al., 2023). In contrast, regular physical activity, such as resistance and impact exercises, improves bone remodeling and enhances bone mineralization, bone mineral density (BMD), and structural properties. This improves balance and postural stability and ultimately reducing the risk of falls, osteoporotic fractures, and related functional impairments (Kistler-Fischbacher et al., 2021; Lyu et al., 2024; Cai et al., 2023). The mechanisms through which exercise improves bone metabolism involve numerous regulatory factors.

Silent information regulator of transcription 1 (SIRT1) is a member of the sirtuin family of nicotinamide adenine dinucleotide (NAD^+^)-dependent class III histone deacetylases. These molecules catalyze the deacetylation of protein substrates during NAD^+^-mediated cleavage (Canto and Auwerx, 2012). SIRT1 regulates multiple biological processes, such as glucose and lipid metabolism, immune response, and endocrine function, in various tissues, including muscle, adipose tissue, and liver (Nogueiras et al., 2012; Huang et al., 2021; Qiang et al., 2011; Chang and Guarente, 2014). Studies have increasingly highlighted the significance of SIRT1 in bone metabolism. SIRT1 is implicated in the regulation of bone homeostasis and prevention of bone-related disorders. SIRT1 expression is reduced in the femoral neck of osteoporotic patients and is negatively correlated with sclerostin, an inhibitor of bone formation (El-Haj et al., 2016). Consistently, SIRT1 knockout mice exhibit a low bone mass phenotype. Highlighting the regulatory role of SIRT1 in bone density (Zainabadi et al., 2017). Notably, high-intensity and repeated exercise training stimulate an increase in SIRT1 expression. In response to the mechanical stress induced by exercise, SIRT1 plays a pivotal role in regulating both bone formation and resorption (Zhu et al., 2023; Kauppinen et al., 2013; Juan et al., 2023). Here, we review the mechanisms by which SIRT1 modulates bone metabolism through various direct and indirect biological processes.

## 2 SIRT1 directly regulates bone metabolism

Bone health is maintained through a delicate balance between bone formation, mediated by osteoblasts, and bone resorption, regulated by osteoclasts. Bone marrow mesenchymal stem cells (BMSCs), which are multipotent stem cells capable of differentiating into osteoblasts, adipocytes, and chondrocytes (Fu et al., 2019). The stable differentiation of BMSCs into osteoblasts promotes bone formation, while differentiation into adipocytes competitively inhibits it (Caplan and Bruder, 2001; Gao et al., 2023). Therefore, regulating the adipogenesis/osteogenesis ratio is essential in bone metabolism. Fortunately, some studies have shown that mechanical movement not only promotes osteogenic differentiation, but also reduces the adipogenic/osteogenic ratio and promotes bone formation (Liu et al., 2024; Shi et al., 2022).

Osteoclasts originate from the monocyte/macrophage lineage of hematopoietic stem cells. An increase in osteoclastogenesis enhances bone resorption, contributing to decreased bone density (Boyle et al., 2003). External factors such as estrogen deficiency and aging can disrupt bone homeostasis by affecting processes such as inflammation, autophagy, oxidative stress, and aging, ultimately affecting bone formation and resorption and triggering metabolic bone diseases such as osteoporosis (Curtis et al., 2015; O'Brien, 1996). Exercise reduces the levels of the receptor activator of nuclear factor-kappa B ligand (RANKL), a key membrane-bound factor involved in osteoclast differentiation, thereby reducing osteoclast activity and inhibiting bone resorption (Shi et al., 2022).

SIRT1 influences exercise-regulated osteoblast and osteoclast formation. For example, low-magnitude vibration training inhibits the SIRT1/p53/p21 pathway, alleviates osteoblast senescence, and subsequently improves bone architecture of osteoporosis, thereby reducing bone loss and fracture incidence (Wen et al., 2021; Bianchi et al., 2022). Additionally, interval running downregulates RANKL expression in bone marrow macrophages, thereby reducing osteoclast formation and bone resorption. This effectively alleviate bone loss caused by menopause and promotes high SIRT1 expression, which regulates the BMP signaling pathways to enhance osteogenic differentiation and bone formation (Kim et al., 2019). The mechanisms by which exercise regulates SIRT1 and influences bone metabolism are outlined in the following sections.

### 2.1 Inflammation

Inflammation has a profound effect on bone metabolism. During the early acute phase of inflammation, inflammatory macrophages (M1) increase the expressions of pro-inflammatory factors such as tumor necrosis factor alpha (TNF-α), IL-1, and IL-6. These factors enhance osteoclast differentiation and activity while inhibiting osteoblast differentiation (Munoz et al., 2020). In contrast, during the later chronic inflammatory phase, M1 macrophages polarize into tissue-regenerative macrophages (M2) that secrete growth factors that promote BMSC-mediated bone formation and bone remodeling (Murray and Wynn, 2011; Mosser and Edwards, 2008). However, under conditions of estrogen deficiency or aging, the failure of M1 to M2 polarization leads to the activation of RANKL and NF-κB-mediated osteoclast differentiation and bone resorption (Yao et al., 2021).

Exercise is integral in regulating the inflammatory response, preventing and reducing osteoporosis (Zhang et al., 2022). Appropriate exercise increases the secretion of anti-inflammatory cytokines, such as IL-2, IL-10, IL-12, and interferon, which support bone formation, while reducing the secretion of proinflammatory cytokines like IL-1 (Yuan et al., 2016; Zhang et al., 2022). Interestingly, high-intensity exercise can induce osteoarthritis. For example, increasing speed running (80 min/day, 5°, maximum speed 20 m/min for 60 min) promotes the level of proinflammatory factor TNF-α and reduces the levels of cartilage structural proteins COL2A and ACAN. In contrast, moderate-intensity running (40 min/day, constant speed of 8 m/min) attenuates cartilage inflammation, reduces the level of proinflammatory factor TNF-α, and results in less morphological damage to articular cartilage (as indicated by the Mankin score), along with a higher density of subchondral trabeculae (Cao et al., 2022). These findings suggest that while excessive exercise may harm bone and joints, exercise of appropriate intensity is beneficial to bone and joint health.

Exercise modulates SIRT1 expression and helps mitigate inflammation in diseases. For example, 8 weeks moderate-intensity treadmill and swimming training (1 h/day) activated SIRT1, leading to a reduction in proinflammatory factors such asIL-1β, IL-6, and TNF-α, as well as NF-κB-p65, thereby alleviating tissue damage and improving the dysfunction in type 2 diabetes mellitus mice (Lang et al., 2020; Habibi et al., 2022). Remarkably, activated SIRT1 promotes M2 macrophage formation, inhibits NF-κB and TNF-α levels, and accelerates the resolution of the inflammatory response. This, in turn, reduces osteoclast differentiation, survival and bone resorption, helping to maintain BMD (Kauppinen et al., 2013; Ye et al., 2022; Park et al., 2023).

### 2.2 Aging

Aging is a complex process that affects the stability of various physiological functions, including those of the skeletal and endocrine system, and leads to diseases such as osteoporosis, osteoarthritis, and sarcopenia (Safiri et al., 2020). The initial stage of aging is cellular senescence, which results from DNA damage and mitochondrial dysfunction (He et al., 2023). This process leads to cell cycle arrest, reduced activity of mesenchymal cells, and a decline in bone turnover, thereby impairing bone formation and enhancing bone resorption, ultimately contributing to bone fragility (Samsonraj et al., 2023; Falvino et al., 2024). During cellular senescence, autophagic activity, energy metabolism regulation, stress resistance, and metabolic status decline (Chen et al., 2020). Aging of BMSCs leads to functional and structural degeneration, reduced proliferative capacity, diminished osteogenic differentiation, increased adipogenic differentiation, and exacerbated bone–fat imbalance (Qadir et al., 2020; Zhang et al., 2011). Concurrently, the aging of osteoblasts further contributes to a reduction in bone formation and promotes bone aging, bone loss, and hinders skeletal healing and recovery (Lin et al., 2019).

Exercise upregulates the expression of SIRT1 in aging mice. Treadmill exercise, for example, upregulates SIRT1 expression in the bones of aging mice (Zhu et al., 2023). SIRT1 activation plays a key role in regulating BMSCs, bone collagen, and bone capillaries. The expression of SIRT1 is downregulated in aged BMSCs. Overexpression of SIRT1 has been shown to delay MSC aging without impairing their adipogenic and osteogenic potential. In contrast, knockdown of SIRT1 slows BMSCs growth and accelerates cellular aging, primarily through the activation of the aging marker P16 (Yuan et al., 2012; Ozturk et al., 2009). Furthermore, SIRT1 knockout mice exhibit a 50% reduction in cortical bone density and display significantly reduced biomechanical strength (Artsi et al., 2022). Specifically, the activation of SIRT1 leads to deacetylation and subsequent inhibition of Kremen1, a senescence-specific surface marker, which mitigates the aging of BMSCs and restores the self-repair capacity of aged bones (Wang et al., 2019; Chen et al., 2020). Additionally, SIRT1 regulates the osteogenic and adipogenic differentiation of BMSCs, promoting bone formation and suppressing fat accumulation, thereby delaying bone loss in aging mice (Song et al., 2019). Moreover, SIRT1 exerts anti-senescence effects on osteoblasts by inhibiting the degradation of bone matrix collagen, enhancing osteoblast activity, reducing DNA damage, and minimizing the accumulation of harmful substances such as cadmium (Zhang et al., 2013; Zhou et al., 2023). Notably, exercise also enhances angiogenesis, improves blood supply to the musculoskeletal system, and increases capillary density through the upregulation of SIRT1, which in turn boosts exercise endurance and performance (Das et al., 2018).

### 2.3 Autophagy

Autophagy is a metabolic process that selectively degrades and recycles damaged or aged organelles to maintain cell homeostasis and survival. Autophagy is essential for maintaining stem cell stemness and differentiation capacity (Garcia-Prat et al., 2016; Wang et al., 2020). Mitophagy promotes intracellular homeostasis by reducing intracellular reactive oxygen species (ROS) produced by damaged mitochondria and by generating ATP during degradation processes (Golden et al., 2010). In bone tissue, Autophagy determines the regenerative potential and function of BMSCs by providing the energy and metabolic precursors required for differentiation (Ceccariglia et al., 2020; Wang et al., 2023). Autophagy also regulates osteoblast differentiation and mineralization by interacting with signaling and transcription factors such as BMP and ATF4 (Wang et al., 2023; B'Chir et al., 2013; Yang et al., 2021).

Running exercise enhances autophagy levels in osteoporotic and type 2 diabetic mice, as evidenced by increased levels of the autophagy marker LC3II and improved bone density (Zhu et al., 2023; Chen et al., 2021). SIRT1-mediated autophagy improves cellular function of bone cells (Wang et al., 2021; Ye et al., 2022; Kim et al., 2022). Mechanical strain upregulate both the mRNA and protein expression of SIRT1, increasing autophagy and enhancing osteogenic differentiation of BMSCs. SIRT1 knockdown diminishes autophagy level in BMSCs by stretch stimulation, demonstrating that mechanical stimuli promote autophagy in BMSCs via SIRT1 activation, thereby regulating osteogenic differentiation (Zhu et al., 2023). Furthermore, activated SIRT1 protected osteoblast activity and differentiation from dexamethasone and fluoride damage by deacetylation of FoxO1 and its target substrates Rab7 and Bnip3, as well as the PI3K/Akt/mTOR pathway (Yang et al., 2019; Gu et al., 2016).

### 2.4 Oxidative stress

Oxidative stress generates ROS that regulate cell behavior, extracellular matrix comp osition, and bone tissue architecture (Cerqueni et al., 2021). Various factors, including unbalanced diet, smoking, and genetic predisposition, increase ROS levels, exacerbate oxidative stress, suppress osteoblast activity and differentiation, induce apoptosis, and compromise bone mineralization and osteogenesis (Cerqueni et al., 2021; Bai et al., 2004). H_2_O_2_-induced oxidative stress enhances adipogenic markers such as PPARγ2 and LEPTIN, and downregulates osteogenic factors such as RUNX2 and COL1A, thereby increasing the fat/bone ratio and weakening the osteogenic effect of BMSCs (Lin et al., 2018).

Exercise training restores the abnormal redox balance and downregulates the expression of hypoxia-inducible factor-1 alpha, a key regulatory factor under hypoxic conditions, thereby alleviating bone degeneration (Sierra-Ramirez et al., 2022). Swimming activates SIRT1/AMPK signaling and upregulates the antioxidant gene NRF2, leading to a reduction in pathological ROS overproduction (Zou et al., 2021). Activated SIRT1 regulates osteoclast and bone-fat imbalance. One study revealed that SIRT1 inhibits oxidative stress-related pathways in bone by deacetylating p66Shc, thereby suppressing the ROS/NF-κB signaling pathway in response to high glucose and palmitate-induced osteoclast differentiation (Qu et al., 2018). Whilst, Resveratrol-induced SIRT1 activation inhibits excessive adipogenesis, improves adipogenic/osteogenic imbalance, promotes bone formation by deacetylation of FOXO3a, the ROS-sensitive transcription factor, and increasing RUNX2 transcription (Lin et al., 2018; Tseng et al., 2011).

## 3 SIRT1 indirectly regulates bone metabolism

In addition to its direct effects on the skeleton, physical activity exerts indirect regulatory effects on bone metabolism through various systems, including the skeletal muscle, nervous system, immune system and endocrine system (Yuan and Song, 2022). For example, exercise promotes myostatin, interleukin-6, Irisin, and apelin in muscles to enter the blood, allowing them to act on bones and maintain the balance between bone absorption and bone formation (Zhao et al., 2023). Among these interactions, SIRT1 is a crucial regulatory factor in the crosstalk (Dolan et al., 2020).

### 3.1 Muscle–bone interactions

A biomechanical connection exists between skeletal muscle and bone. The bone responds to the mechanical load generated by muscle contraction and adapts its mass and structure to meet the demands of movement. Skeletal muscle fibers, also known as skeletal muscle cells, are large, multinucleated cells. In individuals with sarcopenia, decreased muscle fiber volume and function lead to reduced mechanical bone loading, resulting in bone loss (Frost, 2003; Trajanoska et al., 2019). Conversely, effective muscle contraction provides specific mechanical load delivery to the bones in terms of frequency, rate, amplitude, and distribution, thereby stimulating bone growth (Hsieh and Turner, 2001; Hart et al., 2017). Moderate-to high-intensity exercise, such as progressive resistance and impact exercise training significantly improved muscle strength and BMD (O'Bryan et al., 2022; Ng et al., 2023). However, low-intensity aerobic exercise and swimming (less than 3 h/week) had no effect on BMD (Abe et al., 2022; Su et al., 2020). Exercise-induced expression of SIRT1 in muscle cells enhances muscle contraction by increasing the number of myonuclei and size of the myonuclear domain (Radak et al., 2020; Ross et al., 2018). In addition, regular exercise can promote cell proliferation by increasing SIRT1 expression in satellite cells, restoring the regenerative capacity of aged muscle and promoting the repair of muscle damage (Myers et al., 2019).

Exercise accelerates muscle energy metabolism. Increased energy expenditure during physical activity increases ATP demand, resulting in elevated levels of NAD^+^ (White and Schenk, 2012). Notably, decreased NAD^+^ levels in aging or sarcopenic populations can exacerbate physiological decline (Mills et al., 2016). However, aerobic exercise restores NAD^+^ levels, potentially delaying the progression of related diseases (De la Rosa et al., 2020; Hu et al., 2021; Wu et al., 2023; Mills et al., 2016). The increase in NAD^+^ levels provides a substrate for SIRT1, which promotes mitochondrial biogenesis through both PGC-1α-dependent and -independent pathways, thereby regulating energy metabolism, DNA damage repair, muscle cell differentiation, and functionality (White and Schenk, 2012; Koltai et al., 2010).

By regulating mitochondrial homeostasis, exercise enhances muscle function. Intense physical activity activates SIRT1, leading to increased mitochondrial biogenesis and oxidative capacity, which, in turn, improves the content and functionality of skeletal muscle mitochondria (Vargas-Ortiz et al., 2019; Williams and Gurd, 2012). Furthermore, SIRT1 activation maintains the vitality of soleus muscle mitochondria, even under the microgravity of space flight, by oxidizing palmitoylcarnitine. This activation also improves soleus muscle mass and maximum contraction, attenuates muscle atrophy, and ultimately alleviates declining femoral BMD and strength (Momken et al., 2011). Interestingly, there seems to be a gender difference in SIRT1 secretion by muscle. After 6 months of high-intensity aerobic weight loss exercise in people over 50 years old, SIRT1 expression in the vastus lateralis increased, and women were higher than men (Ryan and Li, 2023). Another study showed that after an acute session of sprint interval training, there was no gender difference in the increase of SIRT1 after exercise (Dun et al., 2024), which may be related to the type and duration of exercise. Women have a stronger ability to oxidize fat, which gives them a unique advantage in long-term endurance exercise (Maher et al., 2009).

Through biochemical stimulation, exercise accelerates muscle function. Myokines, such as myostatin and irisin, which are products of muscle secretion, also transmit signals between muscle and bone through endocrine pathways (Herrmann et al., 2020; Zhao et al., 2023). Intermittent running promotes muscle mass and growth in rats with sarcopenia by upregulating the muscle growth factor myogenin, and enhancing SIRTs (Kim et al., 2019). Irisin, a cleavage product of fibronectin type III domain-containing protein 5 (FNDC5), is a well-known muscle-secreted peptide whose expression increases during exercise and is positively correlated with bone mechanical properties (Tenorio et al., 2017; Liu et al., 2021; Zhang et al., 2020). The injection of irisin into mice induces skeletal muscle hypertrophy and enhances muscle strength. This effect is presumably mediated by alleviating skeletal muscle atrophy through muscle stem cell activation and enhanced protein synthesis (Reza et al., 2017). A study of resistance exercise in mice with myocardial infarction suggested that irisin improves myocardial fibrosis by activating the AMPK-SIRT1 pathway. Based on these findings, we infer that SIRT1 plays a vital role in the crosstalk between exercise and the muscle–bone interface.

### 3.2 Nerve–bone interactions

Bone metabolism is directly controlled by the central nervous system. Various central neurohormones, neuropeptides, neurotransmitters, and their intracellular signaling pathways regulate bone metabolism (Huang et al., 2019). Brain-derived neurotrophic factor (BDNF) is widely distributed in the mammalian brain where it regulates neuronal differentiation and dopaminergic neurotransmission (Bi et al., 2024). It is an instructive mediator of central nervous system functional and structural plasticity and is essential for neuronal maintenance, support, and regeneration; it participates in memory synthesis and consolidation (Colucci-D'Amato et al., 2020; Allen et al., 2013).

BDNF regulates osteoblast differentiation, elicits new bone formation, and facilitate fracture healing in bone cells (Ida-Yonemochi et al., 2017; Kilian et al., 2014). Furthermore, BDNF facilitates the osteogenic differentiation of BMSCs by binding to the TrkB receptor and phosphorylates downstream Erk1/2 (Liu et al., 2018). Exercise promotes BDNF expression through SIRT1 activation. For example, an 8-week treadmill exercise regimen was shown to activate SIRT1 and regulates the PGC-1α/FNDC5/BDNF signaling pathway, resulting in increased BDNF concentration and decreased levels of pro-inflammatory cytokines IL-1β, IL-6, and TNF-α in the hippocampus of mice. These changes ultimately enhanced BDNF release and improved cognitive impairments (Lang et al., 2020). Similarly, 1 month of voluntary running wheel exercise induced BDNF expression through SIRT1-dependent upregulation of PGC-1α (El Hayek et al., 2019). Based on these findings, we deduce that exercise promotes bone metabolism by enhancing neural–bone–muscle regulation mediated by SIRT1.

## 4 Conclusion

**FIGURE 1:**
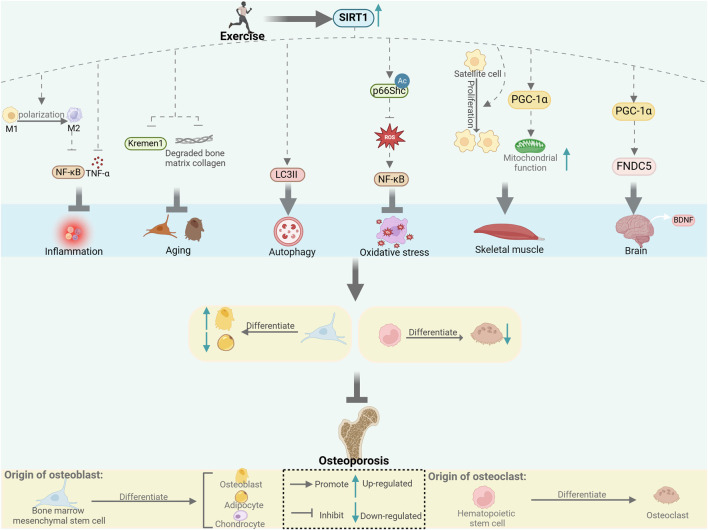
Exercise affects bone metabolism through SIRT1. SIRT1 regulates bone metabolism through inflammation, aging, autophagy and oxidative stress, and through muscle-bone and nerve-bone crosstalk. [This figure was created using BioRender (https://BioRender.com)].

The close connection between SIRT1 and exercise, as well as its impact on bone metabolism, suggests that SIRT1 influences bone metabolism through multiple mechanisms during exercise, including biological processes such as inflammation, aging, autophagy, and oxidative stress, and through the crosstalk between muscle–bone and nerve–bone interactions (Figure 1). We also summarize the different forms and doses of exercise that improve bone metabolism through SIRT1 (Table 1). Since there are limited studies on the direct regulation of SIRT1 by exercise to improve bone metabolism, this review is mostly indirect analysis and summary. The minor number of animal experiments is also a limitation of this article. Based on our findings, we hypothesized that SIRT1 is an emerging exercise effector with potential for regulating bone health. Further research on the specific mechanism of SIRT1-mediated exercise regulation of bone health will help to formulate more reasonable exercise prescriptions and develop effective strategies to prevent and improve bone metabolic diseases such as osteoporosis.

**TABLE 1:** Various forms of exercise improve bone metabolism through SIRT1.

Subject		Exercise	Intensity	Duration	Underlying mechanism	Results	Reference
Animal	OVX rat	Interval running on treadmill	Low intensity: 16 m/min, 0°; high intensity: 23 m/min, 15°; alternate between 3 min high and 2 min low intensity for 5 cycles	30 min/day, 5 days/week, 6weeks	Inflammation↓ Antioxidant↑	Bone loss↓ osteoclast formation↓ bone formation↑	Kim et al. (2019)
	Aging mouse	Gradually increasing intensity running on treadmill	Speed starts at 15 m/min, increased by 1.5 m/min/week to 25 m/min, 0°	Time starts at 20 min, increased by 5 min/week to 55 min (5 days/week, 8 weeks)	Autophagy ↑	Osteoblastogenesis ↑	Zhu et al. (2023)
	Aging rat	Vertical whole-body vibration	0.3 g, 90 Hz.	30 min/day, 5 day/week, 12 weeks	Senescence↓ SIRT1/p53/p21↑	Bone formation↑	Wen et al. (2021)
Cell	BMSC	Mechanical stretch	4% deformation, 0.5 Hz.	4 h/day, 7 days	Autophagy↑	Osteogenic differentiation↑	Zhu et al. (2023)
	Osteogenic cell	Low magnitude vibration	0.3 g, 90 Hz.	30min/day, 5 days	Osteogenic cells senescence↓		Wen et al. (2021)
